# Comparison of percutaneous cryosurgery and surgical resection for the treatment of small hepatocellular carcinoma

**DOI:** 10.3892/ol.2013.1314

**Published:** 2013-04-19

**Authors:** ZHIWEI LI, CHUNHUI ZHANG, CHANGJIE LOU, FEIHU YAN, YINLING MAO, XUAN HONG, YANQIAO ZHANG

**Affiliations:** 1 Department of Gastrointestinal Medical Oncology, The Third Affiliated Hospital of Harbin Medical University, Harbin, Heilongjiang 150081, P.R. China; 2 Cancer Detection and Prevention Institute of Harbin Medical University, Harbin, Heilongjiang 150081, P.R. China

**Keywords:** hepatocellular carcinoma, cryosurgery, surgical resection, overall survival, recurrence-free survival

## Abstract

The present study aimed to compare the outcome of percutaneous cryosurgery (PC) with surgical resection (SR) in the treatment of solitary, small hepatocellular carcinoma (HCC), by performing a retrospective cohort study on 82 patients with solitary HCCs who had received either PC (24 patients) or SR (58 patients). All patients underwent pretreatment blood chemistry tests and an imaging evaluation and were regularly followed up with blood and radiological tests following treatment at The Third Affiliated Hospital of Harbin Medical University, Harbin, Heilongjiang, China. The primary endpoint was overall survival (OS) and the secondary endpoints were those of recurrence-free survival (RFS) and adverse events. In the study, the one-, three- and five-year OS rates following surgery were 100, 75.00 and 66.67%, respectively, in the PC group, and 100, 77.59 and 70.69%, respectively, in the SR group. The corresponding RFS rates at one, three and five years after PC and SR were 83.33, 45.83 and 29.17%, respectively, in the PC group and 84.48, 48.28 and 32.76%, respectively, in the SR group. There were no significant differences between these two groups in terms of OS and RFS. There were also no significant differences between the two groups in terms of OS and RFS when comparing the patients with liver cirrhosis (LC) in the PC group (n=16) and the patients with LC (n=39) in the SR group. No significant factors were identified in the multivariate analysis of the risk factors contributing to OS and RFS. Although there were no statistically significant differences between the two groups in terms of the rate of serious adverse events (P=0.82), the incidence of serious adverse events in the SR group was noticeably higher compared with the PC group. Moreover, the duration of hospitalization in the SR group was significantly longer compared with the PC group (P<0.01). These results suggested that PC is as effective as SR in the treatment of solitary, small HCC, while being less invasive, with a shorter duration of hospitalization and a reduction in patient expenditure compared with SR. Thus, PC may be the first choice for the treatment of solitary, small HCC.

## Introduction

Hepatocellular carcinoma (HCC) is the fifth most common type of cancer worldwide and the third most common cause of cancer mortality (1,2). The prognosis of HCC is generally poor. It represents a major public health problem in the Asia-Pacific region, where the incidence of viral hepatitis is high. The incidence of HCC in China alone accounts for 55% of all cases worldwide (3). By emphasizing the importance of HCC surveillance in patients with chronic liver disease in endemic Asian countries, the treatment of small HCC has become a focus in hepatobiliary surgery. Surgical resection (SR) is widely accepted as a curative treatment for the majority of patients with small HCC who are unwilling to receive liver transplantations (4,5). SR remains the best hope for a cure, but is suitable for only 9–27% of patients (6,7). The presence of significant background liver cirrhosis (LC) often precludes hepatic resection in patients with HCC. Recurrence in the liver remnant is also common in patients who have undergone radical hepatic resection (8).

Local ablative techniques, including percutaneous ethanol injection, microwave coagulation therapy, radiofrequency ablation and percutaneous cryosurgery (PC) have become increasingly popular in the treatment of small HCC due to the severity of the underlying liver disease. PC, also known as cryosurgery, is a technique based on the *in situ* freezing and devitalization of tissues, which may be applied and controlled precisely to produce a predictable zone of necrosis that destroys the target lesion, as well as an appropriate margin of surrounding tissue (9). In China, PC has been widely used to ablate lung, liver and kidney cancer due to its ease of use, safety, cost-effectiveness and minimal invasiveness. Previous studies have shown PC to give good results from the perspective of tumor control, and PC with ultrasound (US) guidance or computed tomography (CT) monitoring is feasible, safe and effective for the treatment of HCC (9,10). However, debate continues with regard to whether PC or SR is the most suitable therapy for small HCC. In the present study, a retrospective cohort study was conducted to compare the results of PC and SR in the treatment of small HCC.

## Materials and methods

### 

#### Characteristics of study population

Between June 2005 and July 2011, 82 patients with solitary HCCs ≤3 cm in diameter received curative treatment using PC or SR at the Third Affiliated Hospital of Harbin Medical University (Harbin, Heilongjiang, China). Prior to performing PC or SR, a full discussion was held between the physician and surgeon. Subsequent to being provided with enough information, including the contents of the discussion between the physician and surgeon, the patients themselves made the decision as to whether they received PC or SR. PC was administered to 24 patients and SR to 58 patients. Written informed consent was obtained from all patients. The protocols for PC and SR were approved by the ethics committee of the Third Affiliated Hospital of Harbin Medical University. The present study consisted of a retrospective analysis of patient records. All treatments were performed in an open-label manner. The primary end point was overall survival (OS) and the secondary end points were recurrence-free survival (RFS) and adverse events. The characteristics of the study population are shown in Table I.

#### HCC diagnosis

HCC was diagnosed using abdominal US and dynamic CT scans (hyper-attenuation during the arterial phase in all or certain areas of the tumor and hypo-attenuation in the portal-venous phase) and/or magnetic resonance imaging (MRI), mainly based on the recommendations of the American Association for the Study of Liver Diseases (11). Arterial and portal phase dynamic CT images were obtained at ∼30 and 120 sec, respectively, following the injection of the contrast material. With regard to the diagnosis of LC, a specimen resected at surgery was used for the SR group and a biopsy specimen was used for the PC group. The baseline characteristics of the two groups are shown in Table I. There were no significant differences between the two groups, with the exception of platelet count (P=0.02).

#### Equipment

The cryosurgery equipment was an 8-cryoprobe surgery system manufactured by Endocare Corporation (Irvine, CA, USA), with superconducting cryoprobes 2, 3 and 5 mm in diameter. US diagnostic apparatus (GE Healthcare, Milwaukee, WI, USA) and high-speed 16-slice spiral CT (Philips, Amsterdam, The Netherlands) were used for probe guidance and localization.

### Treatment

#### Cryosurgery

**Figure 1.**
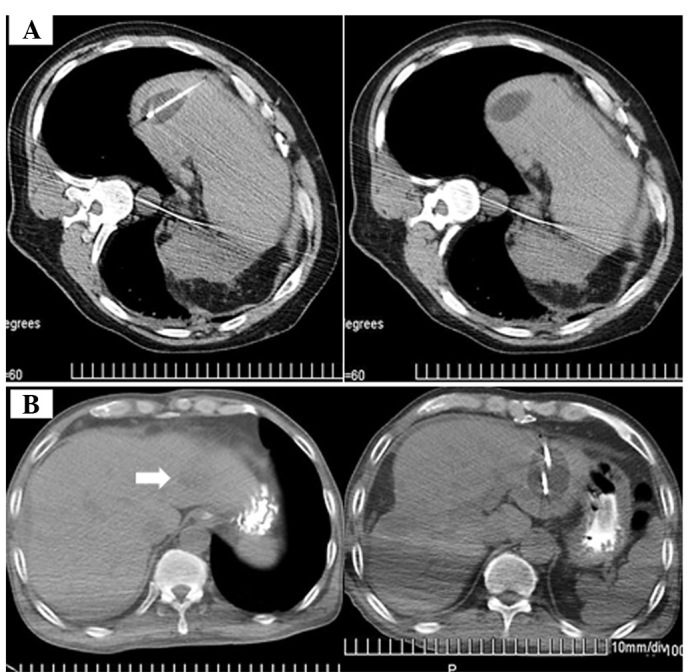
(A) A 45-year-old male with small HCC. Left, cryoprobe and ice-ball formation; and right, the cryoablation area. (B) A 52-year-old male with small HCC. Left, tumor (arrow); and right, cryoprobes and ice-ball formation. HCC, hepatocellular carcinoma.

Cryosurgery was performed in a CT room. Following routine disinfection and local anesthesia, a 0.5-cm incision was created through the planned puncture spot into the skin and subcutaneous tissue. US and CT were used to place the guide needle into the tumor tissue in the liver. The cryoprobes were then introduced into the tumor using a sheath-and-guidewire technique. The cryoprobes were stabilized and the sheath was withdrawn by 3–5 cm. The procedure was repeated as additional cryoprobes were required. Once all the cryoprobes had been placed, the cryosurgery system was initiated to begin rapid freezing. The temperature of the cryoprobes was decreased to −100°C within 1 min. The temperature was then gradually decreased and maintained between −150°C and −160°C for 20 min. The ice-ball size was monitored during the freezing process and technical success was defined at the point when the extension of a visible ice ball was beyond 5 mm from the tumor margin. Once the freezing cycle was complete, the heating system was initiated to re-warm the cryoprobes and the secondary cycle was repeated. Once the freezing and re-warming cycles were complete, the cryoprobes were withdrawn and a hemostatic gelatin sponge was used to stop bleeding and fill the sinuses. Sterile gauze was used to cover and dress the wounds and an abdominal bandage was used to fix the gauze. Representative cryosurgery images are shown in Fig. 1.

*SR.* SR was performed under general anesthesia using a right subcostal incision with a midline extension. An anatomical partial hepatectomy was performed with a resection margin of ≥1 cm over the tumor, based on intraoperative ultra-sonography (IOUS) guidance. IOUS was routinely performed to estimate the location, size and number of vessels feeding the tumor, as well as to produce an exact vascular map of the liver anatomy. When liver function approached normal and any adverse events had disappeared following surgical resection, patient discharge was permitted.

#### Evaluation and follow-up

All patients were evaluated by examining serum tumor markers (α-fetoprotein, AFP), liver function markers [alanine aminotransferase (ALT) and aspartate aminotransferase, (AST)] and total bilirubin (T-Bil), as well as tumor changes as visualized with CT or MRI prior to and following surgery. The primary endpoint was OS. The secondary endpoints were those of RFS and adverse events.

#### Statistical analysis

The levels of serum tumor and liver function markers were compared prior to and following treatment using a t-test and χ^2^ tests. OS was calculated from the date of enrollment to the date of mortality or last follow-up. The OS and RFS curves were generated using the Kaplan-Meier method and compared using the log-rank test. The relative prognostic significance of the variables for predicting OS was assessed using univariate and multivariate Cox proportional hazards regression models. All variables with P<0.05 as evaluated using univariate analysis were subjected to multivariate analysis. The results of the multivariate analysis are presented as the hazard ratio (HR) with the corresponding 95% confidence interval (CI). All statistical analyses were performed using SPSS software (version 17.0, SPSS Inc., Chicago, IL, USA). All tests were two-sided and P<0.05 was considered to indicate a statistically significant difference.

## Results

### 

#### Patients

All patients completed the treatment and follow-up. There were no surgery-related mortalities. Ultimately, 24 patients were included in the PC group and 58 patients were included in the SR group. In addition, no patients succumbed within the hospitalization period, making the mortality rate 0% for the two groups.

#### Patient OS and RFS

**Figure 2.**
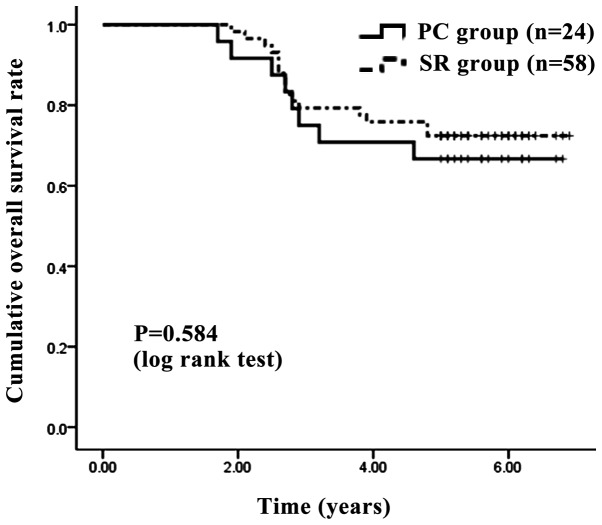
Cumulative OS rate. The one-, three- and five-year OS rates after surgery were 100, 75.00 and 66.67%, respectively, in the PC group and 100, 77.59 and 70.69%, respectively, in the SR group. No significant difference was observed between the two groups, as determined using the log-rank test (P=0.584). OS, overall survival; PC, percutaneous cryosurgery; SR, surgical resection.

**Figure 3.**
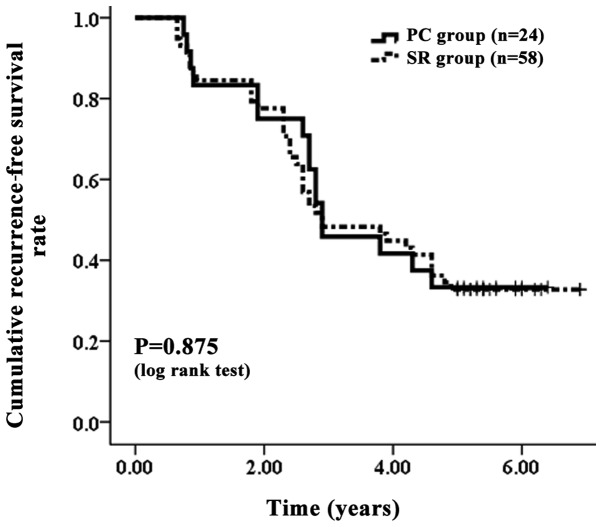
Cumulative RFS rate. The corresponding RFS rates at one, three and five years after PC and SR were 83.33, 45.83 and 29.17%, respectively, in the PC group and 84.48, 48.28 and 32.76%, respectively, in the SR group. No significant differences were observed between the two groups, as determined using the log-rank test (P=0.875). RFS, recurrence-free survival; PC, percutaneous cryosurgery; SR, surgical resection.

The median follow-up periods were 4.7 years (range, 1.7–6.4 years) in the PC group and 4.9 years (range, 2.4–6.5 years) in the SR group. In the PC group, eight patients (33.33%) succumbed during the follow-up period. The causes of mortality were all due to HCC recurrence. In the SR group, 17 patients (29.31%) succumbed during the follow-up period. The causes of mortality were HCC recurrence (15 patients) and miscellaneous causes (two patients). The one-, three- and five-year OS rates following PC and SR were 100, 75.00 and 66.67%, respectively, in the PC group and 100, 77.59 and 70.69%, respectively, in the SR group (Fig. 2). The corresponding RFS rates at one, three and five years after PC and SR were 83.33, 45.83 and 29.17%, respectively, in the PC group and 84.48, 48.28 and 32.76%, respectively, in the SR group (Fig. 3). In terms of OS (P=0.584) and RFS (P=0.875), no significant differences were observed between the two groups.

#### Patient OS and RFS with or without LC

**Figure 4.**
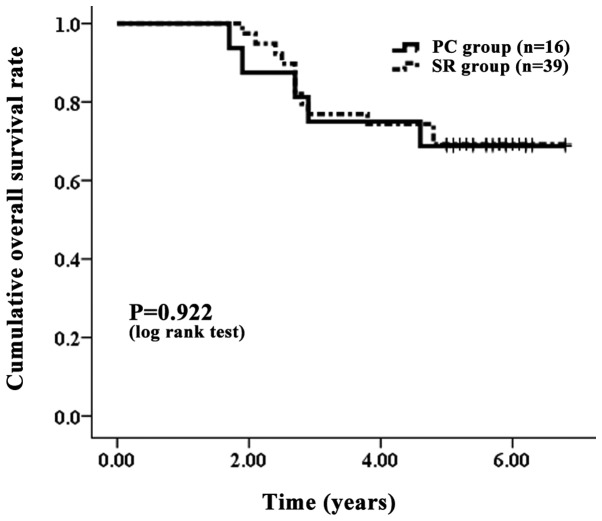
Cumulative OS rate with LC. The PC group contained 16 patients with LC and the SR group contained 39 patients with LC. The one-, three- and five-year OS rates were 100, 75.00 and 68.75%, respectively, in the PC group with LC and 100, 76.92 and 66.67%, respectively, in the SR group with LC. No significant differences were observed between the two groups, as determined using the log-rank test (P=0.992). OS, overall survival; LC, liver cirrhosis; PC, percutaneous cryosurgery; SR, surgical resection.

**Figure 5.**
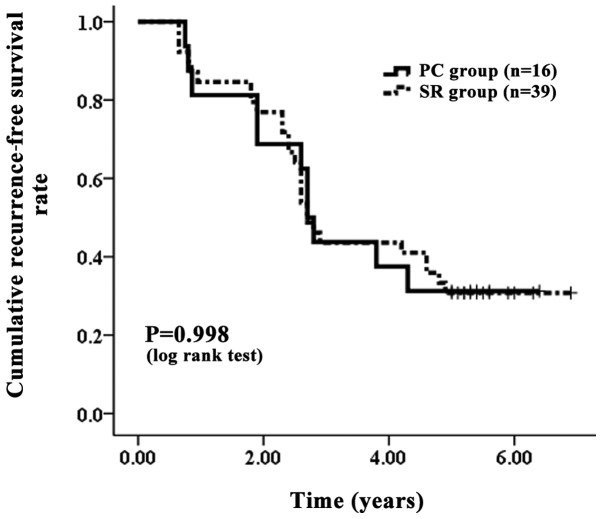
Cumulative RFS with LC. The PC group contained 16 patients with LC and the SR group contained 39 patients with LC. The corresponding RFS rates at one, three and five years after PC and SR were 81.25, 43.75 and 31.25%, respectively, in the PC group with LC and 84.62, 43.59 and 30.77%, respectively, in the SR group with LC. No significant differences were observed between the two groups, as determined using the log-rank test (P=0.998). RFS, recurrence-free survival; LC, liver cirrhosis; PC, percutaneous cryosurgery; SR, surgical resection.

**Figure 6.**
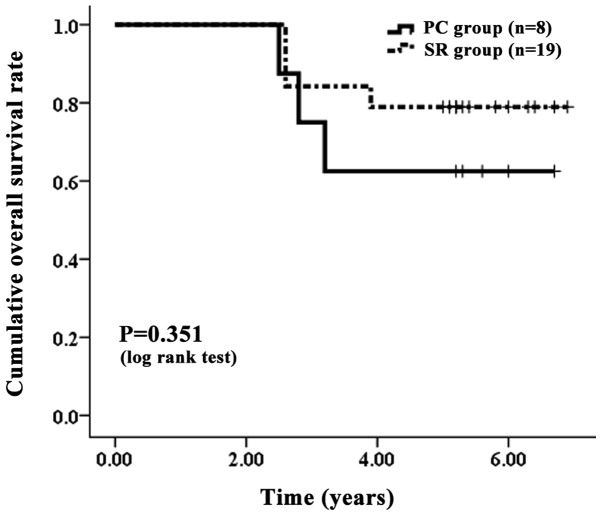
Cumulative OS rate without LC. The PC group contained eight patients with LC and the SR group contained 19 patients with LC. The one-, three- and five-year OS rates were 100, 75.00 and 62.50%, respectively, in the PC group without LC and 100, 78.95 and 78.95%, respectively, in the SR group without LC. No significant differences were observed between the two groups, as determined using the log-rank test (P=0.351). OS, overall survival; LC, liver cirrhosis; PC, percutaneous cryosurgery; SR, surgical resection.

**Figure 7.**
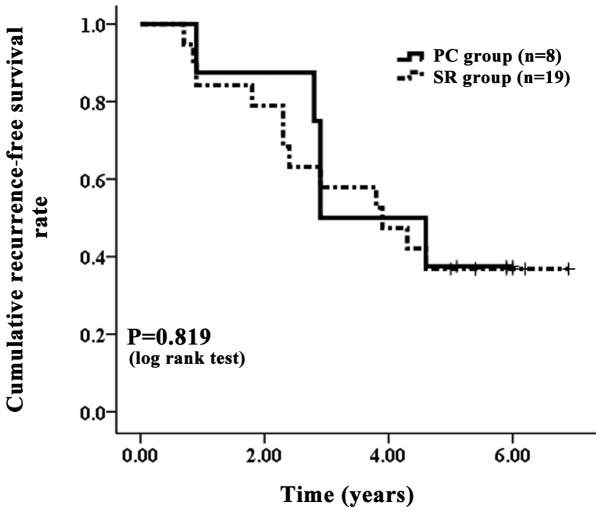
Cumulative RFS rate without LC. The PC group contained eight patients with LC and the SR group contained 19 patients with LC. The corresponding RFS rates at one, three and five years after PC and SR were 87.50, 50.00 and 37.50%, respectively, in the PC group without LC and 84.2, 57.89 and 36.84%, respectively, in the SR group without LC. There were no significant differences between these two groups, as determined using the log-rank test (P=0.819). RFS, recurrence-free survival; LC, liver cirrhosis; PC, percutaneous cryosurgery; SR, surgical resection.

Comparisons were made between PC and SR group patients with LC. There were 16 PC group patients and 39 SR group patients with LC. The one-, three- and five-year OS rates were 100, 75.00 and 68.75%, respectively, in the PC group with LC and 100, 76.92 and 66.67%, respectively, in the SR group with LC (Fig. 4). The corresponding RFS rates at one, three and five years after PC and SR were 81.25, 43.75 and 31.25%, respectively, in the PC group with LC and 84.62, 43.59 and 30.77%, respectively, in the SR group with LC (Fig. 5). In terms of OS (P=0.922) and RFS (P=0.998) with LC, no significant differences were observed between the two groups. The PC and SR group patients without LC were also compared. There were 8 PC group patients and 19 SR group patients without LC. The one-, three- and five-year OS rates were 100, 75.00 and 62.50%, respectively, in the PC group without LC and 100, 78.95 and 78.95%, respectively, in the SR group without LC (Fig. 6). The corresponding RFS rates at one, three and five years were 87.50, 50.00 and 37.50%, respectively, in the PC group without LC and 84.21, 57.89 and 36.84%, respectively, in the SR group without LC (Fig. 7). In terms of the OS (P=0.351) and RFS (P=0.819) of the patients without LC, no significant differences were observed between the two groups.

#### Univariate and multivariate analysis of prognostic factors contributing to OS and RFS

In the univariate analysis of factors contributing to OS, serum albumin (P=0.047) and platelet count (P=0.041) were observed to be significant factors (Table II). However, in the multivariate analyses involving these two factors, no factors were significant factors contributing to OS. Similarly, in the univariate analysis of factors contributing to RFS, only platelet count (P=0.014) was identified as a significant factor (Table III). However, in the multivariate analyses involving this factor, it was also not a significant factor contributing to RFS.

#### Serious adverse events

Serious adverse events were more frequent in the SR group (5/58, 8.62%) compared with the PC group (1/24, 4.17%), although no statistically significant differences were identified between these two groups (P=0.820; Table IV). The serious adverse events in the SR group were as follows: bile leakage (two patients); refractory ascites (one patient); acute pulmonary embolism (one patient); and intra-abdominal bleeding (one patient). The only serious adverse event in the PC group was intra-abdominal bleeding (one patient).

#### Duration of hospitalization

The duration of hospitalization was significantly longer in the SR group (15.2±9.7 days) compared with the PC group (8.6±4.3 days; P<0.01; Table IV).

## Discussion

The present study demonstrated that PC is a reliable, safe and minimally invasive method that may be used to treat small HCC. Firstly, the OS and RFS rates were the same for patients with solitary HCCs ≤3 cm in diameter treated with either PC or SR. Secondly, the results showed that PC had certain advantages over SR, being less invasive, causing less serious adverse events and resulting in a shorter hospitalization period. Thirdly, ultrasound and CT were simultaneously applied to guide the cryoprobes in the present study, which greatly improved the precision of probe localization, reducing the damage to the normal liver tissue and vasculature, and thus enhancing the safety of the therapy.

Patients with cirrhosis are at high risk of developing malignant disease and US is recommended every six months. Surveillance with US allows a diagnosis at the early stages when the tumor may be curable by resection, liver transplantation or ablation and five-year survival rates >50% may be achieved (12). LC is an important prognostic factor of HCC. However, with regard to the OS and RFS of patients with LC, no differences were observed between the two groups in the present study. Moreover, in the univariate and multivariate analysis of factors contributing to OS and RFS, LC was not identified as a significant prognostic factor. A possible reason for this may be that all patients who underwent surgery had improved liver function (the Child-Pugh classification of each patient was A or B).

It has been reported that patients with small, solitary tumors and well-preserved liver function are the most suitable candidates for surgical resection (12). Liver transplantation is the most beneficial approach for individuals who are not good candidates for resection. However, donor shortages greatly limit its applicability. Percutaneous ablation is frequently used for treatment but its effectiveness is limited by tumor size and localization (12). Partial hepatectomy is considered the gold standard therapy, with the aim of delivering a cure, in patients with resectable HCC who have normal liver function and are in a good general condition (13). It has become possible to reduce perioperative mortality to <5% depending on the extent of resection and hepatic reserve (14). The improved outcome is primarily as a result of advances in surgical and radiological techniques, perioperative care and more cautious patient selection (15).

The present long-term follow-up data suggest that PC and local SR achieve similar survival rates in patients with small HCC, although a previous study has reported that PC reduces mortality rates in cancer patients (16). There is a general consensus that a complete response to PC therapy in patients is associated with an improved outcome. Therefore, in the present study, patients with HCC tumors ≤3 cm in size were selected. Moreover, the study also observed that there were no significant differences between the two treatment groups in terms of OS and RFS. One possible reason for this is that a sufficient ablative margin was made around the tumor by PC, which may have suppressed invasion by micro-dissemination (Fig. 1). Consequently, obtaining a sufficient ablative margin around the tumor appears to be essential in PC therapy. However, the present study had several limitations. Firstly, it was a retrospective cohort study. Patients who had a good hepatic reserve tended to receive SR and this may have led to bias. Secondly, the present study was limited to patients who had undergone curative treatment. These problems should be resolved in future prospective studies.

In summary, PC was demonstrated to be as effective as SR in the treatment of patients with solitary, small HCC who had undergone curative treatment, while being less invasive and resulting in shorter hospitalization periods compared with SR. Cryosurgery caused minimal damage to the normal liver tissue and resulted in few serious adverse events. In addition, cryosurgery has also been shown to reduce the risk of disseminating malignant cells (17), which may be attributed to the procedures ability to completely necrotize local tumor tissue. Therefore, PC should be considered for future application in the treatment of solitary, small HCC.

The present study has clinical significance, providing optimized individualized treatment selection for patients with solitary, small HCC.

## Figures and Tables

**Table I.** Patient characteristics.

Variable	PC group (n=24)	SR group (n=58)	P-value, T or χ^2^
Mean age, years (range)	514±10	58±9	0.08^a^
Gender			0.68^b^
Male	16 (66.67)	33 (56.90)	
Female	8 (33.33)	25 (43.10)	
AFP (ng/ml)	321±661.78	127±370.62	0.36^a^
Liver cirrhosis			0.21^b^
With cirrhosis	16 (66.67)	39 (67.24)	
Without cirrhosis	8 (33.33)	19 (32.76)	
Liver function (Child-Pugh classification)			0.30^b^
Class A	19 (79.16)	45 (77.59)	
Class B	5 (20.83)	13 (22.41)	
Tumor size (cm)	2.25±0.56	2.35±0.49	0.09^a^
Serum albumin (g/dl)	3.89±0.72	3.78±0.51	0.13^a^
Total bilirubin (mg/dl)	0.75±0.41	0.87±0.54	0.12^a^
Platelet count (×10^4^/mm^3^)	10.92±5.74	13.11±3.89	0.02^a^
ALT (U/l)			
>40	14 (58.33)	42 (72.41)	1.60^b^
≤40	10 (41.67)	16 (27.59)	
AST (U/l)			0.76^b^
>40	2 (50.00)	36 (62.07)	
≤40	12 (50.00)	22 (37.93)	

All data are presented as mean ± standard deviation or no. of patients (%) unless otherwise indicated.

a T-test;

b χ^2^-test. PC, percutaneous cryosurgery; SR, surgical resection; ALT, alanine aminotransferase; AST, aspartate aminotransferase; AFT, α-fetoprotein

**Table II.** Univariate and multivariate analysis of the prognostic factors contributing to OS.

Variables	Univariate analysis	Multivariate analysis	
	P-value	Hazard ratio (95% CI)	P-value
PC vs. SR	0.370	0.927 (0.82–1.273)	0.685
Gender (male vs. female)	0.750		
Age (>65 vs. ≤65 years)	0.520		
LC vs. non-LC	0.063		
Tumor size (>2 vs. ≤2 cm)	0.740		
Serum AFP (>100 vs. ≤100 ng/ml)	0.490		
Serum albumin (>100 vs. ≤100 g/dl)	0.047	1.223 (0.675–2.334)	0.263
Platelet count (>10 vs. ≤10×10^4^/mm^3^)	0.041	1.117 (0.539–2.183)	0.588
T-Bil (>1.0 vs. ≤1.0 mg/dl)	0.260		

OS, overall survival; PC, percutaneous cryosurgery; SR, surgical resection, LC, liver cirrhosis; AFP, α-fetoprotein; T-Bil, total bilirubin; CI, confidence interval.

**Table III.** Univariate and multivariate analysis of the prognostic factors contributing to RFS.

Variables	Univariate analysis	Multivariate analysis	
	P-value	Hazard ratio (95% CI)	P-value
PC vs. SR	0.312	0.964 (0.873–1.153)	0.738
Gender (male vs. female)	0.525		
Age (>65 vs. ≤65 years)	0.415		
LC vs. non-LC	0.023	0.766 (0.412–1.168)	0.157
Tumor size (>2 vs. ≤2 cm)	0.461		
Serum AFP (>100 vs. ≤100 ng/ml)	0.697		
Serum albumin (>100 vs. ≤100 g/dl)	0.097		
Platelet count (>10 vs. ≤10×10^4^/mm^3^)	0.014	1.238 (0.783–1.918)	0.295
T-Bil (>1.0 vs. ≤1.0 mg/dl)	0.573		

RFS, recurrence-free survival; PC, percutaneous cryosurgery; SR, surgical resection; LC, liver cirrhosis; AFP, α-fetoprotein; T-Bil, total bili-rubin; CI, confidence interval.

**Table IV.** Hospitalization duration and serious adverse events.

Variables	PC group (n=24)	SR group (n=58)	P-value, T or χ^2^
Duration of hospitalization (days)	8.6±4.3	15.2±9.7	<0.01^a^
Serious adverse events, n (%)	1 (4.17)	5 (8.62)	0.82^b^

Data are presented as mean ± standard deviation unless stated otherwise.

a T-test;

b Fisher’s exact test. PC, percutaneous cryosurgery; SR, surgical resection.

## Abbreviations:

HCC: hepatocellular carcinoma;
PC: percutaneous cryosurgery;
SR: surgical resection;
OS: overall survival;
RFS: recurrence-free survival;
LC: liver cirrhosis;
ALT: alanine aminotransferase;
AST: aspartate aminotransferase;
AFP: α-fetoprotein;
T-Bil: total bilirubin;
US: ultrasound;
CT: computed tomography;
MRI: magnetic resonance imaging
